# The journey of Diagnostic Pathology in 2009, perspectives for 2010

**DOI:** 10.1186/1746-1596-4-47

**Published:** 2009-12-22

**Authors:** Klaus Kayser

**Affiliations:** 1 UICC-TPCC, Institute of Pathology, Charite, Berlin, Germany

## Abstract

Editorial: The journey of Diagnostic Pathology in 2009, perspectives for 2010

## Editorial

*Diagnostic Pathology *is approaching the end of its fourth year of publication. From the beginning, *Diagnostic Pathology *has operated as an independent scientific journal, allowing the Editor-in-Chief and Editorial Board to control the content of the journal, while the publisher provides support in these activities and regulates other aspects including production and layout of the published articles. The separation of these activities allows the Editorial team to focus on the content and direction for the journal, making it the best home for research in the area.

In order to make *Diagnostic Pathology *open access, the journal levies an article-processing charge (APC) for each article accepted for publication. These costs are necessary to continue to provide the production and hosting services as well as additional support, but they remain competitive compared to other open access journals. Many funders support open access by allowing their grants to be used to cover the cost of publishing in open access journals [1] and more institutions are becoming BioMed Central Members, or Supporter Members, to help their researchers to publish open access. However, it still remains a burden for authors, who have to both complete a scientifically reliable and interesting publication and pay for it. It is therefore important that authors feel that the costs of open access publication offer a beneficial return. How can the return be judged? What is the nature of the return, and how does it benefit the authors?

This is primarily a question to the authors, although it is also of importance to the journal Editors. Publishing an article usually requires research investigation which can last for months or years. Results and interpretation are the fruits that are offered to colleagues, and "should be eaten". They have to be "sweet" and to "fit into the taste" of the potential readers. Every successful merchant knows that the wrapping of an article is as important as the content that has to be sold successfully. The main interest of an author in publishing scientific results is a broad access to the corresponding scientific world, and a high reputation of the article (at home and in the scientific environment). Also of interest are the technical publication procedures, such as fast, helpful, non-biased, and reliable review of the article, concrete assistance in improving the paper, and fast publication of the accepted final version.

How does *Diagnostic Pathology *deal with these reimbursements?

Open access journals, i.e., those that can be read for free, have a wide reader community. The readers are not limited to colleagues who are working within the same field of the published article. They often visit a journal in search for new ideas or even by chance, for example by coming across relevant articles in PubMed or PubMed Central. In addition, open access journals are embedded in an electronic communication medium that offers several advantages for a fast and "screening-like" visit. They are all provided with a fast search function, adequate links to "related articles", easy to copy functions, or "post a comment" message. *Diagnostic Pathology *offers all these items. With a wide readership, articles are well accessed. A total of six articles were published in November 2009, two of which have already been read by more than 400 colleagues. These articles were submitted to *Diagnostic Pathology *at the end of August 2009, and published in November 2009, i.e., the time taken from submission, to review, revise and final publication amounts to less than three months. The average time of acceptance was calculated as 74 days (from submission to publication).

In addition to our standard scientific quality levels it is also our policy to encourage and support colleagues working in developing countries to publish their findings in our journal. Taking out randomly 10 published articles in 2009, two of them were submitted from the USA, one article each from Brazil, China, Egypt, Germany, Iran, Poland, Spain and Turkey. These figures indicate that *Diagnostic Pathology *is truely a world-wide and well known scientific journal already in its fourth year of publication.

The scientific quality of a journal can be partly judged by statistical methods such as the impact factor or H-index. Although it is questionable whether these factors reflect to the reputation of a scientific journal in its community, an impact factor is often mandatory if scientists want to publish their results in the selected journal. The main disadvantage of the impact factor is its absolute value, i.e. it depends upon the size of the journal specific scientific community. The impact factor of pathology journals is basically low in comparison to that of molecular biology or biochemistry journals.

*Diagnostic Pathology *received its first official impact factor in 2009, of 1.11 and is now included in the journal citation index. At a starting point the value 1.11 can be regarded as high. It is already above that of several long term established scientific journals in surgical pathology.

Both figures, namely the submission of articles written in all parts of the world, the frequent access of interested colleagues, and the newly established citation index contribute to the journal's reputation, and can be considered as a high quality return for the invested time and financial input of the authors.

In summary, a successful year for *Diagnostic Pathology *is coming to its end despite all constraints. A big step forward in reputation and notification has been achieved, which to our satisfaction benefits our authors.

We want to express our deep gratitude to, and we wish all our authors, readers, reviewers, and our publication team a Merry Christmas and a Happy and Healthy New Year.

Klaus Kayser

Editor in Chief
